# Neural Basis of Working Memory Enhancement after Acute Aerobic Exercise: fMRI Study of Preadolescent Children

**DOI:** 10.3389/fpsyg.2016.01804

**Published:** 2016-11-21

**Authors:** Ai-Guo Chen, Li-Na Zhu, Jun Yan, Heng-Chan Yin

**Affiliations:** ^1^College of Physical Education, Yangzhou UniversityYangzhou, China; ^2^School of Physical Education and Sports Science, Beijing Normal UniversityBeijing, China

**Keywords:** acute aerobic exercise, working memory, N-back, brain activation patterns, fMRI, preadolescent children

## Abstract

Working memory lies at the core of cognitive function and plays a crucial role in children’s learning, reasoning, problem solving, and intellectual activity. Behavioral findings have suggested that acute aerobic exercise improves children’s working memory; however, there is still very little knowledge about whether a single session of aerobic exercise can alter working memory’s brain activation patterns, as assessed by functional magnetic resonance imaging (fMRI). Therefore, we investigated the effect of acute moderate-intensity aerobic exercise on working memory and its brain activation patterns in preadolescent children, and further explored the neural basis of acute aerobic exercise on working memory in these children. We used a within-subjects design with a counterbalanced order. Nine healthy, right-handed children were scanned with a Siemens MAGNETOM Trio 3.0 Tesla magnetic resonance imaging scanner while they performed a working memory task (N-back task), following a baseline session and a 30-min, moderate-intensity exercise session. Compared with the baseline session, acute moderate-intensity aerobic exercise benefitted performance in the N-back task, increasing brain activities of bilateral parietal cortices, left hippocampus, and the bilateral cerebellum. These data extend the current knowledge by indicating that acute aerobic exercise enhances children’s working memory, and the neural basis may be related to changes in the working memory’s brain activation patterns elicited by acute aerobic exercise.

## Introduction

Working memory involves temporary storage and manipulation of information assumed necessary for a wide range of complex cognitive activities (Baddeley, 1992, 2003). It is an essential element for learning, memory, decision-making, cognitive control, other high-level cognitive activities, and brain development (Baddeley, 1992, 2003; Ericsson and Kintsch, 1995; Bechara et al., 1998; de Jong, 1998; Passolunghi and Siegel, 2001; Swanson and Sachse-Lee, 2001; Bull et al., 2008). Deficits in working memory will seriously harm the development of children’s physical, mental, and social achievements; conversely, individuals, local communities, and society will benefit from well-developed working memory (Siegel and Ryan, 1989; Ericsson and Kintsch, 1995; Bechara et al., 1998; de Jong, 1998; Passolunghi and Siegel, 2001; Bull et al., 2008). Although working memory has a neural anatomical basis, it is flexible and plastic, and thus, can be improved through training, especially in high correlation with children’s cognitive development (Olesen et al., 2004; Westerberg and Klingberg, 2007; Takeuchi et al., 2010; Kamijo et al., 2011; Mrazek et al., 2013). Therefore, working memory has attracted increasing research attention in various fields and has become a frontier in interdisciplinary research. Identification of effective methods to develop children’s working memory is a focus of the current research.

A burgeoning body of literature has emerged on exercise’s positive effects on the brain and cognition. Aerobic exercise as an effective method for improving children’s brain and cognitive function has been gradually recognized and practiced (Davis et al., 2007, 2011; Erickson and Kramer, 2009; Diamond and Lee, 2011; Erickson et al., 2011; Chang et al., 2012, 2013; Chapman et al., 2013; Hötting and Röder, 2013; Verburgh et al., 2013; Chen et al., 2014). Recent findings have also suggested that acute aerobic exercise enhances working memory (Pontifex et al., 2009; McMorris et al., 2011; Li et al., 2014). Nevertheless, it is not clear whether the neural basis of improvement in children’s working memory is elicited by acute aerobic exercise.

Functional magnetic resonance imaging (fMRI) non-invasively and safely measures and maps brain activity (Fox and Raichle, 2007). With fMRI, brain activation can be evaluated by measuring the blood oxygenation level-dependent (BOLD) contrast signal, which reflects a change in the ratio of oxygenated to deoxygenated hemoglobin that occurs with brain activation and increases in local blood volume. In a growing number of studies, fMRI is applied to directly understand how brain function changes with aerobic exercise or training (Kramer and Erickson, 2007; Chaddock et al., 2010; Davis et al., 2011; Erickson et al., 2011; Li et al., 2014). Through fMRI, working memory’s neural basis has been found to have a specific pattern of brain activation, which, in working memory, mainly includes frontal and parietal cortices (Cohen et al., 1997; Klingberg et al., 2002; Baddeley, 2003; Klingberg, 2006; Darki and Klingberg, 2014; Ester et al., 2015; Harding et al., 2015). These brain regions’ functional specialization and cooperation are the operating basis of working memory (Diwadkar et al., 2000; Baddeley, 2003). Moreover, studies from cognitive psychology and neuroscience have revealed that working memory training increases and decreases in task-related BOLD activity in different regions associated with increases in working memory capacity (Constantinidis and Klingberg, 2016). That is, both increases and decreases in the BOLD signal can be informative about the stimulus maintained in working memory, reflecting excitatory and suppressive responses to stimuli’s orientation and motion. Additionally, a decrease in the BOLD signal in a certain area is often interpreted as an increase in the area’s “efficiency” in performing its function. In short, improvement in working memory relates to its activation pattern changes (Klingberg et al., 2002; Olesen et al., 2004; Westerberg and Klingberg, 2007; Takeuchi et al., 2010; Kamijo et al., 2011; Mrazek et al., 2013). Therefore, the key to clarifying the neural basis of working memory enhancement caused by children’s acute aerobic exercise is to reveal changes in working memory’s brain activation patterns elicited by acute aerobic exercise.

In summary, the present study explores the effect of acute moderate-intensity aerobic exercise on working memory and its brain activation patterns in preadolescent children, and further explored the neural basis of acute aerobic exercise on working memory in these children. We hypothesize that our study would both replicate previous studies, demonstrating that acute aerobic exercise improves working memory, and extend our current understanding of this process to discover that acute aerobic exercise can better optimize working memory’s brain activation pattern.

## Materials and Methods

### Ethic Statement

The study protocol was approved by the Institutional Review Board of Beijing Normal University. All participants and their guardians provided written consent, and the protocol was approved by the institutional review board of Beijing Normal University.

### Participants

Nine healthy children in fifth grade (10 years old; five males, four females) were recruited through primary school advertising. They had normal or corrected-to-normal vision and were right-handed, as assessed by the Edinburgh Test (Oldfield, 1971). They also completed a set of questions about history of drug abuse or inherited disease and general intelligence (Wechsler Intelligence Scale for Children-IV-Chinese Version, WISC-IV-C) (Zhang, 2009). Exclusions included any medical condition that would limit physical activity or affect study results (including neurological or psychiatric disorders). The study was conducted in accordance with the Declaration of Helsinki. The study protocol was approved by the Institutional Review Board of the Brain Imaging Center of the State Key Laboratory of Cognitive Neuroscience and Learning, Beijing Normal University. Written informed consent was obtained from all participants and their legal guardians after experimental procedures were fully explained.

### Acute Aerobic Exercise Protocol

Exercise was performed on a bicycle ergometer (MONARK 834, Sweden) with moderate intensity, which has been shown to benefit children’s cognition (Hillman et al., 2009; Chen et al., 2014). This study used 60–69% of the predicted maximal heart rate, defined as 220 minus age in years, to determine the exercise intensity target heart rate (American College of Sports Medicine, 2006). Participants spent 2 min cycling to warm up, 30 min exercising at moderate intensity, and finally, 3 min cooling down at a self-determined pace. Heart rate was monitored in real time using a Polar heart rate monitor (RS800XSD, Finland) throughout the acute aerobic exercise protocol, which was led by the same instructor, one-on-one.

### Working Memory Task

**FIGURE 1:**
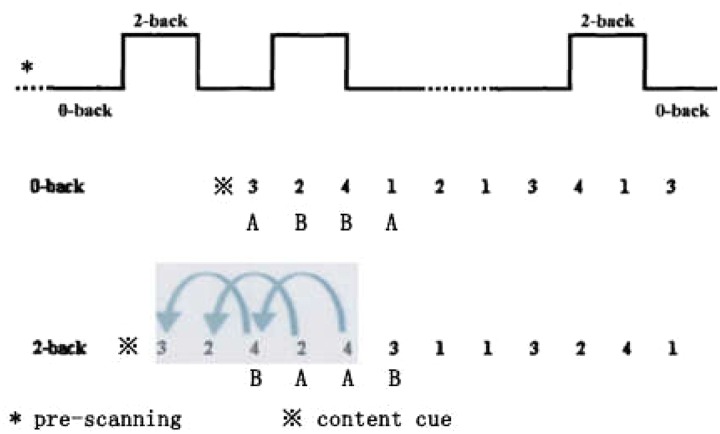
**Design of the N-back working memory task**.

An N-back task (Smith and Jonides, 1997; Owen et al., 2005) was programmed using E-Prime to assess working memory. The task used group block design (**Figure 1**), contained 0- and 2-back conditions, and was performed alternatingly. The 2-back condition was a working memory task, while the 0-back condition was a control task that did not need to manipulate working memory. Thus, the contrast between 0-back and 2-back conditions reflects, to a large degree, time-related aspects of processing stimuli in working memory. The N-back task had five 2-back conditions and six 0-back conditions. The task consisted of a series of changing number stimuli displayed at the center of a computer screen (i.e., 1, 2, 3, 4). The first block of three phases was pre-scanning. Each block of one to four phases was adaptive, and the screen center was “ready,” prompting the participant to prepare to respond. Each stimulus was displayed on the screen for 2,000 ms and each phase’s duration was 2,000 ms. The presentation time for each number stimulus was 1,500 ms, and the reaction time was 500 ms. Working memory’s behavioral performance consisted of mean reaction time (RT) and mean accuracy (Acc); consequently, the shorter the RT, and/or the higher the Acc, the better the participant’s working memory.

Each 0-back condition included 11 phases in random order. The first phase was an experimental content cue: *single* or *double*; when the numeral *1* or *3* appeared, pressing button *A*; when the numeral *2* or *4* appeared, pressing button *B*. These four numbers were displayed randomly at equal probability.

Each 2-back condition included 13 phases in random order. The first phase was an experimental content cue: *Remember and go back 2*; the participant needed to remember the second and third stimuli in order of appearance. When the fourth stimulus appeared, they had to respond whether it was the same as the second; if it was, they pressed *A*, and otherwise, pressed *B* on the keyboard with both hands.

### Experimental Procedure

This experiment had a completely within-subjects design. It was conducted in the Imaging Center for Brain Research at Beijing Normal University. During the first visit, participants and their legal guardians completed all paperwork, including written informed assent/consent, as previously described. Following paperwork completion, an N-back task practice was administered to each participant, and the experimenter checked their performances to ensure that the participants understood the task. If a participant’s task performance was below 80% (Acc), the same practice was re-administered. In the formal experiment, all participants attended two sessions (i.e., baseline and exercise), with the order counterbalanced across participants at the same time on two separate days (a 7-day interval) in which they had not participated in physical education or other structured physical activity. So half participants received the baseline session on the first day and the exercise session on the second day. The other half received the exercise session on the first day and the baseline session on the second day. The baseline session consisted of 30 min of seated rest, during which all participants were fitted with a heart rate monitor and their resting heart rates were recorded. Following the seated rest period, participants completed an N-back task during MRI scanning. The exercise session consisted of a 30-min rest, with the resting heart rate recorded, and an acute aerobic exercise protocol during which HR was recorded in real time. Following the completion of the acute aerobic exercise protocol, once participants’ HRs returned to within 10% of their resting heart rate levels, the N-back task was performed during MRI scanning. Upon completion of both sessions, participants and their legal guardians received fair remuneration for their involvement in the experiment.

### Functional MRI Data Acquisition and Image Processing

Participants underwent one scan for high-resolution structural images of the whole brain on a 3T Siemens Magnetom Trio system (Siemens) with total imaging matrix in the Imaging Center for Brain Research, Beijing Normal University. Functional images were obtained using an echo-planar imaging (EPI) sequence, with the following scan parameters: TR = 2,000 ms, TE = 30 ms, gap = 1 mm, flip angle (FA) = 90°, slice thickness = 3.0 mm, field of view (FOV) = 200 × 200 mm, and inplane resolution = 64 × 64. Resulting data included 148 brain volumes with 33 axial slices. During the fMRI scans, all participants were instructed to stay relaxed and move as little as possible. High-resolution structural images were acquired using a magnetization-prepared rapid gradient echo, three-dimensional T1-weighted sequence (TR = 2000 ms, TE = 3.39 ms, T1 = 1100 ms, FA = 7°, thickness = 1.33 mm, FOV = 200 × 200 mm, acquisition matrix = 256 × 256).

Functional image preprocessing and statistical analyses were conducted with DPABI^1^, based on SPM8^2^. The first three volumes of functional images were discarded for signal equilibrium and participants’ adaptation to scanning noise. Subsequent functional images underwent the following preprocessing steps: slice-timing correction, realignment, co-registration, and New Segment + Diffeomorphic Anatomical Registration Through Exponentiated Liealgebra (DARTEL) with high-resolution structural scans (Ashburner and Friston, 2005). The DARTEL tool (Ashburner, 2007) was used to compute transformations from individual native space to Montreal Neurological Institute (MNI) coordinate space. Then, the segmented BOLD volumes were normalized into standardized MNI space using the DARTEL template and resampled to 3 mm ×3 mm ×3 mm isotropic voxels. Finally, normalized images were smoothed with an 8 mm × 8 mm × 8 mm full width at half maximum Gaussian kernel.

### Statistical Analysis

First, descriptive data were evaluated to determine the appropriateness of exercise intensity manipulation. Second, behavioral improvements in RT and Acc for working memory across the two sessions were analyzed by paired *t*-test with SPSS. Third, functional changes were analyzed with two procedures, individual and group analysis. Individual analysis: The 0-back condition was considered a control task in the present study. The 2-back condition required maintenance and permanent update of relevant pieces of information in working memory. Then, statistical parametric maps (SPMs) were computed for individual participants by using the general linear model (GLM) with separate hemodynamic basis response function modeling MR signal responses for each task period. Contrast images (2-back vs. 0-back) on estimates of interest were obtained for each participant. Group analysis: There were two steps for group analysis based on the contrast images (2-back vs. 0-back). The first was to identify whether the N-back task is successfully inducing the common brain activation patterns of working memory for each session (baseline and exercise), we conducted a one-sample *t*-test on the contrast images (2-back vs. 0-back). The second was to examine neural activation differences between two sessions (baseline and exercise); we conducted a paired *t*-test on the contrast images (2-back vs. 0-back) to detect acute aerobic exercise gains.

## Results

### Exercise Intensity Manipulation

The heart rates for the baseline and exercise sessions were 41.80 and 64.52%, respectively, of the predicted maximal heart rate [*t*(8) = 23.70, *p* < 0.001]. The two sessions’ differing heart rates and percentages of the predicted maximal heart rate suggest that the selected moderate-intensity exercise was appropriate.

### Behavioral Performances

**Table 1** presents detailed behavioral measures based on the two sessions and the N-back conditions. To explore behavioral performance differences between the baseline and exercise sessions, a paired *t*-test was used on RT and accuracy. There were no significant differences between the baseline and exercise sessions on 0-back RT [*t*(8) = 1.37, *p* > 0.05 and Acc *t*(8) = –1.03, *p* > 0.05; 2-back Acc *t*(8) = –0.82, *p* > 0.05]. The 2-back RT of the exercise session showed significant improvement after acute exercise *t*(9) = 2.79, *p* < 0.05, *r*^2^pb = 0.49; that is, shorter RT demonstrated better working memory.

**Table 1:** Children’s behavioral performances on N-back task in baseline and exercise sessions.

Condition	Baseline session		Exercise session	
	ACC (%)	RT(ms)	ACC (%)	RT(ms)
0-back	0.95 ± 0.03	682.80 ± 52.68	0.96 ± 0.03	643.92 ± 73.35
2-back	0.81 ± 0.03	963.13 ± 125.24	0.82 ± 0.03	877.57 ± 53.74

### Brain Activation Differences

**FIGURE 2:**
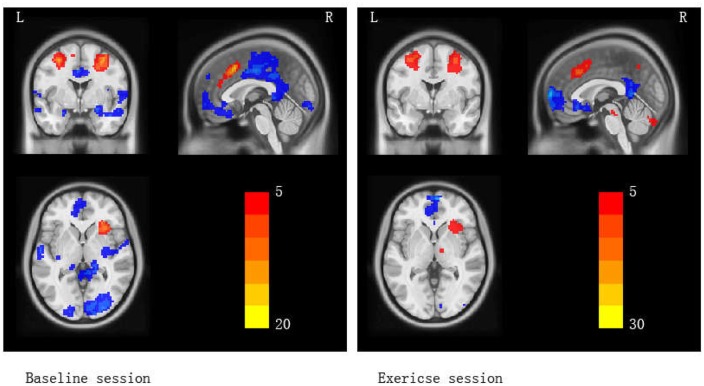
**Brain activation maps (2-back vs. 0-back) for baseline and exercise sessions separately.** Red areas indicate greater activation in 2-back > 0-back. Blue areas indicate lower activation in 2-back > 0-back.

The first analytical goal was to examine working memory activation in two scanning sessions (baseline and exercise) with contrast images (2-back vs. 0-back), calculated as an assessment of the dynamic range of neural differences activation between a control task and a working memory task. The statistical threshold was set at *p* < 0.001 with a cluster size threshold of 75 voxels, which is equivalent to cluster-level *p* < 0.05, AphaSim corrected. Specifically, in the baseline session, regions of significant BOLD activation were the left Superior Frontal Gyrus (SFG), bilateral Middle Frontal Gyrus (MFG), right Inferior Frontal Gyrus (IFG), bilateral Parahippocampa gyrus (PHP), right Middle Occipital Gyrus (MOG), left Superior Temporal Gyrus (STG), and bilateral Cerebellum Posterior Lobe; in the exercise session, right Medial Frontal Gyrus (MEDFG), MFG, left Superior Parietal Lobule (SPL), right Inferior Parietal Lobule (IPL), right Superior Occipital Gyrus (SOG), left Anterior Cingulate Cortex (ACC), right Posterior Cingulate Cortex (PCC), and bilateral Cerebellum Posterior Lobe were activated (**Table 2**; **Figure 2**). This analysis of activated brain regions revealed the common patterns of working memory.

**Table 2:** Children’s brain activation patterns during working memory in baseline and exercise scanning sessions.

Primary regions	Brodmann area	Size (voxel)	Max T-statistic	MNI coordinates		
				* x*	* y*	* z*
Baseline session						
B_Cerebellum Posterior Lobe	–	103	21.84	–36	–60	–45
L_Parahippocampa Gyrus	30	235	–13.23	–24	–21	–18
R_Parahippocampa Gyrus	34	4249	–21.29	24	–3	–15
L_Medial Frontal Gyrus	10	506	–8.62	–3	54	–6
R_Middle Occipital Gyrus	18	1352	–19.21	21	–99	3
L_Superior Temporal Gyrus	48	289	–13.35	–39	–15	15
R_Inferior Frontal Gyrus	47/48	158	17.06	30	18	–9
B_Middle Frontal Gyrus	32/24	844	17.54	0	18	42
L_Superior Frontal Gyrus	9	108	–9.41	–9	48	42
Exercise session						
L_Cerebellum Posterior Lobe	–	111	18.25	–33	–66	–48
B_Cerebellum Posterior Lobe	–	80	9.59	–6	–81	–30
L_Middle Frontal Gyrus	47	84	–9.48	–36	39	–18
L_Anterior Cingulate Cortex	25	131	–9.72	–6	24	–6
R_Medial Frontal Gyrus	10	371	–15.92	6	63	–3
R_Middle Frontal Gyrus	46	246	11.51	45	48	15
R_Superior Occipital Gyrus	19	160	–9.06	33	–84	24
R_Posterior Cingulate Cortex	30	442	–9.49	6	–51	21
L_Middle Frontal Gyrus	32	1015	34.98	–6	21	39
L_Superior Parietal Lobule	7	320	11.94	–6	–72	57
R_Inferior Parietal Lobule	40	215	14.84	48	–42	39

*The statistical threshold was set at p < 0.001, with a cluster size threshold of 75 voxels, which is equivalent to cluster-level p < 0.05, AphaSim corrected. All coordinates are reported in the MNI format.*

**FIGURE 3:**
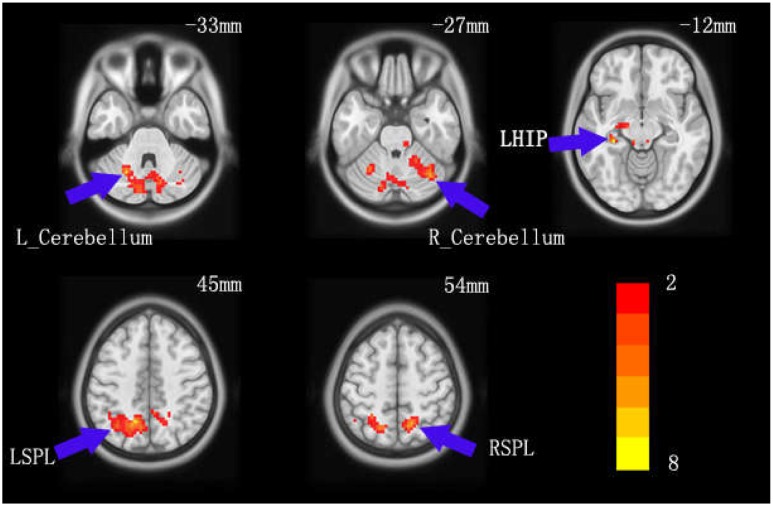
**The change in children’s brain activation pattern of working memory caused by acute aerobic exercise.** Red areas indicate higher activation in the exercise session relative to the baseline session.

In further comparisons of brain regions’ activated changes between the baseline and exercise sessions, a paired *t*-test revealed significant difference. The statistical threshold was set at *p* < 0.025 with a cluster size threshold of 100 voxels, which is equivalent to cluster-level *p* < 0.05, AphaSim corrected. Specifically, the exercise session resulted in greater activation of SPL, IPL, left Hippocampus (HIP), and bilateral Cerebellum (**Table 3**; **Figure 3**). This analysis indicated that acute aerobic exercise significantly increased some regions of brain activities for working memory.

**Table 3:** Significant brain activation of paired *t*-test between children’s baseline and exercise MRI scanning.

Primary regions	Brodmann area	Size (voxel)	Max T- statistic	MNI coordinates		
				*x*	*y*	*z*
L_Superior/Inferior Parietal Lobule	7/40	497	8.64	–15	–57	45
R_Superior Parietal Lobule	5/7	191	6.57	12	–60	54
L_Hippocampus	20	208	8.23	–30	–30	–12
L_Cerebellum	–	347	7.18	–24	–60	–33
R_Cerebellum	–	108	6.47	36	–63	–27

*The statistical threshold was set at p < 0.025, with a cluster size threshold of 100 voxels, which is equivalent to cluster-level p < 0.05, AphaSim corrected. All coordinates are reported in the MNI format.*

## Discussion

This study investigated behavioral and neural effects of children’s acute aerobic exercise on a working memory task. In both baseline and exercise sessions, participants were tested with a working memory task while being scanned. The exercise session, an acute aerobic exercise protocol, was compared to a baseline session without exercise intervention. Consequently, reliable acute aerobic exercise gains emerged, allowing us to test for acute aerobic exercise effects on the two sessions.

### Behavioral Performances

A rapidly growing body of literature indicates that, from both behavioral and neuroelectric perspectives, acute aerobic exercise improves working memory (Lardon and Polich, 1996; Hillman et al., 2008; Erickson and Kramer, 2009; Chang et al., 2012, 2013; Chen et al., 2014; Drollette et al., 2014). As observed here, children’s working memory performance in the exercise session was better than in the baseline session—in agreement with previous studies. Accordingly, the present behavior results have been again verified: acute aerobic exercise beneficially impacts children’s working memory.

### Brain Activation Patterns of Working Memory

To our knowledge, many previous studies have explored the macroscale of working memory’s neural system. First, in an N-back task, the present study successfully induced working memory’s common brain activation patterns. However, how do working memory’s brain activation patterns run? Cognitive neuropsychology observations concluded that working memory’s neural basis is quite widespread, including the cerebral cortex (e.g., frontal, temporal, parietal, and occipital lobes) and subcortical areas (e.g., thalamus, amygdala, hippocampus, and cerebellum) (LaBar et al., 1999; Smith and Jonides, 1999; Diwadkar et al., 2000; Baddeley, 2003; Osaka et al., 2004; Owen et al., 2005). Brain areas in our results matched these and indicated that the N-back task successfully induced working memory activation patterns.

Second, we compared the exercise session’s brain activation maps to those of the baseline session. Regarding acute aerobic exercise-related neural effects, the main findings were that acute aerobic exercise raised working memory’s brain activation patterns in bilateral SPL, left IPL, left HIP, and bilateral cerebellum. These results are fully consistent with our prediction that acute exercise significantly influenced working memory’s brain activities. These results may be of great importance for understanding the neural basis of acute aerobic exercise’s effect on working memory. Indeed, prior studies have demonstrated that improved cognitive function results from brain plasticity changes caused by aerobic exercise (Kramer and Erickson, 2007; Erickson and Kramer, 2009; Voss et al., 2013). One finding reported that high-fitness children showed greater bilateral hippocampal volumes, positively associated with performance on memory task (Chaddock et al., 2010); this study first suggested that aerobic fitness can impact the structure and function of the developing human brain. Similarly, a recent study suggested that significant changes after acute aerobic exercise with brain activation reflected improved working memory in young female college students (Li et al., 2014). Thus, acute aerobic exercise could benefit children’s working memory at a macro-neural level.

What is the meaning of children’s greater brain activation caused by acute aerobic exercise? Several previous studies have addressed this question, assisting us to understand these increases in SPL, IPL, and bilateral cerebellum and hippocampus. Development of functionality in these areas plays an important role in cognitive development during childhood, and parietal cortices are known to be involved in working memory (Jonides et al., 1998; Culham and Kanwisher, 2001; Koenigs et al., 2009). Working memory capacity significantly correlated with brain activities in the parietal gyrus (Vogel and Machizawa, 2004; Todd and Marois, 2005). Similarly, another study reported that higher parietal activity was associated with higher working memory capacity in children (Klingberg et al., 2002). A meta-analysis concluded that the functional topography of the cerebellum is particularly involved in sensorimotor, language, spatial, and working memory (Stoodley and Schmahmann, 2009). Prior evidence indicated that cognitive training could increase neural activity in cerebellar circuits in children with ADHD (Hoekzema et al., 2010). Recent studies of the cerebellum have revealed connections between the cerebellum and parietal gyrus that might support cerebellar contributions to working memory (Allen et al., 2005). The hippocampus plays an important role in the formation of new memories about experienced events (Vinogradova, 2001). Moreover, previous observations found that the parietal lobule is the target of disynaptic output from two subcortical sites, in which the parietal lobule receives a strong disynaptic input from the hippocampus and the cerebellum contains a distinct output channel that targets a portion of the parietal lobule (Clower et al., 2001). At this point, we speculate on the neural basis of improved working memory induced by acute aerobic exercise: Greater activation of the cerebellum might contribute to the parietal cortex’s better functioning and strengthened correlations between the hippocampus and parietal cortex, consequently enhancing children’s working memory.

This study is not without its limitations. First, a completely within-subjects design was used in current study in which all subjects are exposed to every experimental session. The drawback of this design is the absence of control group. However, this design ensures that every subject acts at their own control, so there are few problems with matching age, gender, and lifestyle, reducing the chances of confounding factors. Second, generalization of our results is limited by small sample size. Future studies with a larger sample may be more amenable to investigate the effect of acute moderate-intensity aerobic exercise on working memory and its brain activation patterns in preadolescent children.

## Conclusion

Here, we suggest that acute aerobic exercise results in children’s greater cognitive gains. These data extended the current knowledge base by indicating that acute aerobic exercise enhances children’s working memory, in which the neural basis may be related to changes in working memory’s brain activation patterns elicited by the exercise.

## Author Contributions

A-GC and H-CY designed the study and oversaw the data collection. L-NZ and A-GC analyzed the data and wrote the initial manuscript. JY and H-CY assisted with data analysis and organized the manuscript. All authors played a part in the manuscript’s preparation at each stage of its development. All authors have read and approved the manuscript’s final version.

## Conflict of Interest Statement

The authors declare that the research was conducted in the absence of any commercial or financial relationships that could be construed as a potential conflict of interest.

## Footnotes

1 http://rfmri.org/dpabi

2 http://www.fil.ion.ucl.ac.uk/spm/software/spm8/

## References

[B1] Allen G. McColl R. Barnard H. Ringe W. K. Fleckenstein J. Cullum C. M. (2005). Magnetic resonance imaging of cerebellar-prefrontal and cerebellar-parietal functional connectivity. *Neuroimage* 28 39–48. 10.1016/j.neuroimage.2005.06.013 16023375

[B2] American College of Sports Medicine (2006). *ACSM’s Guidelines for Exercise Testing and Prescription*, 7th Edn. New York, NY: Lippincott Williams and Wilkins.

[B3] Ashburner J. (2007). A fast diffeomorphic image registration algorithm. *Neuroimage* 38 95–113. 10.1016/j.neuroimage.2007.07.007 17761438

[B4] Ashburner J. Friston K. J. (2005). Unified segmentation. *Neuroimage* 26 839–851. 10.1016/j.neuroimage.2005.02.018 15955494

[B5] Baddeley A. (1992). Working memory: the interface between memory and cognition. *J. Cogn. Neurosci.* 4 281–288. 10.1162/jocn.1992.4.3.281 23964884

[B6] Baddeley A. (2003). Working memory: looking back and looking forward. *Nat. Rev. Neurosci.* 4 829–839. 10.1038/nrn1201 14523382

[B7] Bechara A. Damasio H. Tranel D. Anderson S. W. (1998). Dissociation of working memory from decision making within the human prefrontal cortex. *J. Neurosci.* 18 428–437. 9412519 10.1523/JNEUROSCI.18-01-00428.1998 PMC6793407

[B8] Bull R. Espy K. A. Wiebe S. A. (2008). Short-term memory, working memory, and executive functioning in preschoolers: longitudinal predictors of mathematical achievement at age 7 years. *Dev. Neuropsychol.* 33 205–228. 10.1080/87565640801982312 18473197 PMC2729141

[B9] Chaddock L. Erickson K. I. Prakash R. S. Kim J. S. Voss M. W. VanPatter M. (2010). A neuroimaging investigation of the association between aerobic fitness, hippocampal volume, and memory performance in preadolescent children. *Brain Res.* 1358 172–183. 10.1016/j.brainres.2010.08.049 20735996 PMC3953557

[B10] Chang Y. K. Labban J. D. Gapin J. I. Etnier J. L. (2012). The effects of acute exercise on cognitive performance: a meta-analysis. *Brain Res.* 1453 87–101. 10.1016/j.brainres.2012.02.068 22480735

[B11] Chang Y. K. Tsai Y. J. Chen T. T. Hung T. M. (2013). The impacts of coordinative exercise on executive function in kindergarten children: an ERP study. *Exp. Brain Res.* 225 187–196. 10.1007/s00221-012-3360-9 23239198

[B12] Chapman S. B. Aslan S. Spence J. S. DeFina L. F. Keebler M. W. Didehbani N. (2013). Shorter term aerobic exercise improves brain, cognition, and cardiovascular fitness in aging. *Front. Aging Neurosci.* 5:75 10.3389/fnagi.2013.00075 PMC3825180 24282403

[B13] Chen A. G. Yan J. Yin H. C. Pan C. Y. Chang Y. K. (2014). Effects of acute aerobic exercise on multiple aspects of executive function in preadolescent children. *Psychol. Sport Exerc.* 15 627–636. 10.1016/j.psychsport.2014.06.004

[B14] Clower D. M. West R. A. Lynch J. C. Strick P. L. (2001). The inferior parietal lobule is the target of output from the superior colliculus, hippocampus, and cerebellum. *J. Neurosci.* 21 6283–6291. 11487651 10.1523/JNEUROSCI.21-16-06283.2001 PMC6763148

[B15] Cohen J. D. Perlstein W. M. Braver T. S. Nystrom L. E. Noll D. C. Jonides J. (1997). Temporal dynamics of brain activation during a working memory task. *Nature* 386 604–608. 10.1038/386604a0 9121583

[B16] Constantinidis C. Klingberg T. (2016). The neuroscience of working memory capacity and training. *Nat. Rev. Neurosci.* 17 438–449. 10.1038/nrn.2016.43 27225070

[B17] Culham J. C. Kanwisher N. G. (2001). Neuroimaging of cognitive functions in human parietal cortex. *Curr. Opin. Neurobiol.* 11 157–163. 10.1016/S0959-4388(00)00191-4 11301234

[B18] Darki F. Klingberg T. (2014). The role of fronto-parietal and fronto-striatal networks in the development of working memory: a longitudinal study. *Cereb. Cortex* 25 1587–1595. 10.1093/cercor/bht352 24414278

[B19] Davis C. L. Tomporowski P. D. Boyle C. A. Waller J. L. Miller P. H. Naglieri J. A. (2007). Effects of aerobic exercise on overweight children’s cognitive functioning: a randomized controlled trial. *Res. Q. Exerc. Sport* 78 510–519. 10.5641/193250307X13082512817660 18274222 PMC2662758

[B20] Davis C. L. Tomporowski P. D. McDowell J. E. Austin B. P. Miller P. H. Yanasak N. E. (2011). Exercise improves executive function and achievement and alters brain activation in overweight children: a randomized, controlled trial. *Health Psychol.* 30 91–98. 10.1037/a0021766 21299297 PMC3057917

[B21] de Jong P. F. (1998). Working memory deficits of reading disabled children. *J. Exp. Child Psychol.* 70 75–96. 10.1006/jecp.1998.2451 9729450

[B22] Diamond A. Lee K. (2011). Interventions shown to aid executive function development in children 4 to 12 years old. *Science* 333 959–964. 10.1126/science.1204529 21852486 PMC3159917

[B23] Diwadkar V. A. Carpenter P. A. Just M. A. (2000). Collaborative activity between parietal and dorso-lateral prefrontal cortex in dynamic spatial working memory revealed by fMRI. *Neuroimage* 12 85–99. 10.1006/nimg.2000.0586 10875905

[B24] Drollette E. S. Scudder M. R. Raine L. B. Moore R. D. Saliba B. J. Pontifex M. B. (2014). Acute exercise facilitates brain function and cognition in children who need it most: an ERP study of individual differences in inhibitory control capacity. *Dev. Cogn. Neurosci.* 7 53–64. 10.1016/j.dcn.2013.11.001 24309300 PMC6987893

[B25] Ericsson K. A. Kintsch W. (1995). Long-term working memory. *Psychol. Rev.* 102 211–245. 10.1037/0033-295X.102.2.211 7740089

[B26] Erickson K. I. Kramer A. F. (2009). Aerobic exercise effects on cognitive and neural plasticity in older adults. *Br. J. Sports Med.* 43 22–24. 10.1136/bjsm.2008.052498 18927158 PMC2853472

[B27] Erickson K. I. Voss M. W. Prakash R. S. Basak C. Szabo A. Chaddock L. (2011). Exercise training increases size of hippocampus and improves memory. *Proc. Natl. Acad. Sci. U.S.A.* 108 3017–3022. 10.1073/pnas.1015950108 21282661 PMC3041121

[B28] Ester E. F. Sprague T. C. Serences J. T. (2015). Parietal and frontal cortex encode stimulus-specific mnemonic representations during visual working memory. *Neuron* 87 893–905. 10.1016/j.neuron.2015.07.013 26257053 PMC4545683

[B29] Fox M. D. Raichle M. E. (2007). Spontaneous fluctuations in brain activity observed with functional magnetic resonance imaging. *Nat. Rev. Neurosci.* 8 700–711. 10.1038/nrn2201 17704812

[B30] Harding I. H. Yücel M. Harrison B. J. Pantelis C. Breakspear M. (2015). Effective connectivity within the frontoparietal control network differentiates cognitive control and working memory. *Neuroimage* 106 144–153. 10.1016/j.neuroimage.2014.11.039 25463464

[B31] Hillman C. H. Erickson K. I. Kramer A. F. (2008). Be smart, exercise your heart: exercise effects on brain and cognition. *Nat. Rev. Neurosci.* 9 58–65. 10.1038/nrn2298 18094706

[B32] Hillman C. H. Pontifex M. B. Raine L. B. Castelli D. M. Hall E. E. Kramer A. F. (2009). The effect of acute treadmill walking on cognitive control and academic achievement in preadolescent children. *Neuroscience* 159 1044–1054. 10.1016/j.neuroscience.2009.01.057 19356688 PMC2667807

[B33] Hoekzema E. Carmona S. Tremols V. Gispert J. D. Guitart M. Fauquet J. (2010). Enhanced neural activity in frontal and cerebellar circuits after cognitive training in children with attention-deficit/hyperactivity disorder. *Hum. Brain Mapp.* 31 1942–1950. 10.1002/hbm.20988 20336653 PMC6871170

[B34] Hötting K. Röder B. (2013). Beneficial effects of physical exercise on neuroplasticity and cognition. *Neurosci. Biobehav. Rev.* 37 2243–2257. 10.1016/j.neubiorev.2013.04.005 23623982

[B35] Jonides J. Schumacher E. H. Smith E. E. Koeppe R. A. Awh E. Reuter-Lorenz P. A. (1998). The role of parietal cortex in verbal working memory. *J. Neurosci.* 18 5026–5034. 9634568 10.1523/JNEUROSCI.18-13-05026.1998 PMC6792554

[B36] Kamijo K. Pontifex M. B. O’Leary K. C. Scudder M. R. Wu C. T. Castelli D. M. (2011). The effects of an afterschool physical activity program on working memory in preadolescent children. *Dev. Sci.* 14 1046–1058. 10.1111/j.1467-7687.2011.01054.x 21884320 PMC3177170

[B37] Klingberg T. (2006). Development of a superior frontal-intraparietal network for visuo-spatial working memory. *Neuropsychologia* 44 2171–2177. 10.1016/j.neuropsychologia.2005.11.019 16405923

[B38] Klingberg T. Forssberg H. Westerberg H. (2002). Increased brain activity in frontal and parietal cortex underlies the development of visuospatial working memory capacity during childhood. *J. Cogn. Neurosci.* 14 1–10. 10.1162/089892902317205276 11798382

[B39] Koenigs M. Barbey A. K. Postle B. R. Grafman J. (2009). Superior parietal cortex is critical for the manipulation of information in working memory. *J. Neurosci.* 29 14980–14986. 10.1523/JNEUROSCI.3706-09.2009 19940193 PMC2799248

[B40] Kramer A. F. Erickson K. I. (2007). Capitalizing on cortical plasticity: influence of physical activity on cognition and brain function. *Trends Cogn. Sci.* 11 342–348. 10.1016/j.tics.2007.06.009 17629545

[B41] LaBar K. S. Gitelman D. R. Parrish T. B. Mesulam M. M. (1999). Neuroanatomic overlap of working memory and spatial attention networks: a functional MRI comparison within subjects. *Neuroimage* 10 695–704. 10.1006/nimg.1999.0503 10600415

[B42] Lardon M. T. Polich J. (1996). EEG changes from long-term physical exercise. *Biol. Psychol.* 44 19–30. 10.1016/S0301-0511(96)05198-8 8906355

[B43] Li L. Men W. W. Chang Y. K. Fan M. X. Ji L. Wei G. X. (2014). Acute aerobic exercise increases cortical activity during working memory: a functional MRI study in female college students. *PLoS ONE* 9:e99222 10.1371/journal.pone.0099222 PMC4050105 24911975

[B44] McMorris T. Sproule J. Turner A. Hale B. J. (2011). Acute, intermediate intensity exercise, and speed and accuracy in working memory tasks: a meta-analytical comparison of effects. *Physiol. Behav.* 102 421–428. 10.1016/j.physbeh.2010.12.007 21163278

[B45] Mrazek M. D. Franklin M. S. Phillips D. T. Baird B. Schooler J. W. (2013). Mindfulness training improves working memory capacity and GRE performance while reducing mind wandering. *Psychol. Sci.* 24 776–781. 10.1177/0956797612459659 23538911

[B46] Oldfield R. C. (1971). The assessment and analysis of handedness: the Edinburgh inventory. *Neuropsychologia* 9 97–113. 10.1016/0028-3932(71)90067-4 5146491

[B47] Olesen P. J. Westerberg H. Klingberg T. (2004). Increased prefrontal and parietal activity after training of working memory. *Nat. Neurosci.* 7 75–79. 10.1038/nn1165 14699419

[B48] Osaka N. Osaka M. Kondo H. Morishita M. Fukuyama H. Shibasaki H. (2004). The neural basis of executive function in working memory: an fMRI study based on individual differences. *Neuroimage* 21 623–631. 10.1016/j.neuroimage.2003.09.069 14980565

[B49] Owen A. M. McMillan K. M. Laird A. R. Bullmore E. (2005). N-back working memory paradigm: a meta-analysis of normative functional neuroimaging studies. *Hum. Brain Mapp.* 25 46–59. 10.1002/hbm.20131 15846822 PMC6871745

[B50] Passolunghi M. C. Siegel L. S. (2001). Short-term memory, working memory, and inhibitory control in children with difficulties in arithmetic problem solving. *J. Exp. Child Psychol.* 80 44–57. 10.1006/jecp.2000.2626 11511134

[B51] Pontifex M. Hillman C. Fernhall B. O. Thompson K. Valentini T. (2009). The effect of acute aerobic and resistance exercise on working memory. *Med. Sci. Sports Exerc.* 41 927–934. 10.1249/MSS.0b013e3181907d69 19276839

[B52] Siegel L. S. Ryan E. B. (1989). The development of working memory in normally achieving and subtypes of learning disabled children. *Child Dev.* 60 973–980. 10.2307/1131037 2758890

[B53] Smith E. E. Jonides J. (1997). Working memory: a view from neuroimaging. *Cogn. Psychol.* 33 5–42. 10.1006/cogp.1997.0658 9212720

[B54] Smith E. E. Jonides J. (1999). Storage and executive processes in the frontal lobes. *Science* 283 1657–1661. 10.1126/science.283.5408.1657 10073923

[B55] Stoodley C. J. Schmahmann J. D. (2009). Functional topography in the human cerebellum: a meta-analysis of neuroimaging studies. *Neuroimage* 44 489–501. 10.1016/j.neuroimage.2008.08.039 18835452

[B56] Swanson H. L. Sachse-Lee C. (2001). Mathematical problem solving and working memory in children with learning disabilities: both executive and phonological processes are important. *J. Exp. Child Psychol.* 79 294–321. 10.1006/jecp.2000.2587 11394931

[B57] Takeuchi H. Sekiguchi A. Taki Y. Yokoyama S. Yomogida Y. Komuro N. (2010). Training of working memory impacts structural connectivity. *J. Neurosci.* 30 3297–3303. 10.1523/JNEUROSCI.4611-09.2010 20203189 PMC6634113

[B58] Todd J. J. Marois R. (2005). Posterior parietal cortex activity predicts individual differences in visual short-term memory capacity. *Cogn. Affect. Behav. Neurosci.* 5 144–155. 10.3758/CABN.5.2.144 16180621

[B59] Verburgh L. Königs M. Scherder E. J. Oosterlaan J. (2013). Physical exercise and executive functions in preadolescent children, adolescents and young adults: a meta-analysis. *Br. J. Sports Med.* 48 973–979. 10.1136/bjsports-2012-091441 23467962

[B60] Vinogradova O. S. (2001). Hippocampus as comparator: role of the two input and two output systems of the hippocampus in selection and registration of information. *Hippocampus* 11 578–598. 10.1002/hipo.1073 11732710

[B61] Vogel E. K. Machizawa M. G. (2004). Neural activity predicts individual differences in visual working memory capacity. *Nature* 428 748–751. 10.1038/nature02447 15085132

[B62] Voss M. W. Erickson K. I. Prakash R. S. Chaddock L. Kim J. S. Alves H. (2013). Neurobiological markers of exercise-related brain plasticity in older adults. *Brain Behav. Immun.* 28 90–99. 10.1016/j.bbi.2012.10.021 23123199 PMC3544982

[B63] Westerberg H. Klingberg T. (2007). Changes in cortical activity after training of working memory-a single-subject analysis. *Physiol. Behav.* 92 186–192. 10.1016/j.physbeh.2007.05.041 17597168

[B64] Zhang H. (2009). The revision of WISC-IV Chinese version. *Psychol. Sci.* 32 1177–1179.

